# Considerations for skin carcinogenesis experiments using inducible transgenic mouse models

**DOI:** 10.1186/s13104-018-3182-3

**Published:** 2018-01-24

**Authors:** Martyna C. Popis, Rebecca E. Wagner, Fernando Constantino-Casas, Sandra Blanco, Michaela Frye

**Affiliations:** 1 Department of Genetics, University of Cambridge, Downing Street, Cambridge, CB2 3EH UK; 2 Wellcome Trust-Medical Research Council Cambridge Stem Cell Institute, Tennis Court Road, Cambridge, CB2 1QR UK; 3 Department of Veterinary Medicine, Queen’s Veterinary School Hospital, University of Cambridge, Madingley Road, Cambridge, CB3 0ES UK; 4 CIC bioGUNE, Bizkaia Technology Park, 801 Building, 48160 Derio, Spain

**Keywords:** Skin carcinogenesis, Mouse model, Papilloma, SCC, p53, k-ras

## Abstract

**Objective:**

This study was designed to estimate the percentage of non-malignant skin tumours (papillomas) progressing to malignant squamous cell carcinomas (SCCs) in a carcinogenesis study using established transgenic mouse models. In our skin cancer model, we conditionally induced oncogenic point mutant alleles of *p53* and *k*-*ras* in undifferentiated, basal cells of the epidermis.

**Results:**

Upon activation of the transgenes through administration of tamoxifen, the vast majority of mice (> 80%) developed skin papillomas, yet primarily around the mouth. Since these tumours hindered the mice eating, they rapidly lost weight and needed to be culled before the papillomas progressed to SCCs. The mouth papillomas formed regardless of the route of application, including intraperitoneal injections, local application to the back skin, or subcutaneous insertion of a tamoxifen pellet. Implantation of a slow releasing tamoxifen pellet into 18 mice consistently led to papilloma formation, of which only one progressed to a malignant SCC. Thus, the challenges for skin carcinogenesis studies using this particular cancer mouse model are low conversion rates of papillomas to SCCs and high frequencies of mouth papilloma formation.

## Introduction

The most common human cancers arise from epithelia including skin, colon, breast, prostate, or lung, and together cause several million deaths per year [1]. Squamous cell carcinoma (SCC) is the second most common form of skin cancer and predominantly occurs in sun-exposed regions of skin [2]. In other epithelia such as lung and oesophagus, SCCs are often induced by mutagens including tobacco and alcohol or the human papillomavirus (HPV) [3, 4].

Genetic alterations in the *RAS* and *P53* genes are commonly identified as driver genes in aggressive SCCs [5]. To model the human disease, many transgenic mouse cancer models have been generated that accurately recapitulate the genetic alterations found in human tumours. Due to the high frequency of *RAS* mutations in human epithelial cancers, investigations into the role of oncogenes in tumourigenesis commonly induce endogenous *ras* mutations in mice [6]. Endogenous oncogenic *ras* is sufficient to initiate transformation by stimulating proliferation, yet further genetic lesions are required to progress to a malignant tumour [6]. Inactivation of the p53 tumour suppressor is an additional frequent event in tumorigenesis [7]. Activation of both cancer-causing genetic mutations in an inducible fashion in particular cell types of the epidermis is often used to study the cellular and molecular origins of SCCs [8–13].

By applying cancer-inducing genetic alterations to mice, they provide valuable in vivo tumour models to study skin cancer origin, progression, metastasis and chemotherapy resistance. Here, we discuss practical considerations for skin carcinogenesis experiments using inducible transgenic mouse models.

## Main text

### Methods

All transgenic mouse lines in this study are routinely used in carcinogenesis studies and have been described previously: K14-CreER [14], K-ras^LSL-G12D^ [6], and p53^LSL-R172H^ [15]. The lines were obtained from The Jackson Laboratory (https://www.jax.org/). All mice were on a mixed genetic background and age-matched males and females were used. All mice were housed in individually ventilated cages (IVC). The experiments were not performed blinded as the genotype was known to the investigators. However, all mice for breeding and the experiments were chosen randomly.

Tamoxifen was either applied as solution or in form of tamoxifen (free base) pellets (5 or 7.5 mg/pellet) (Innovative Research of America, cat. nr. E-361) were implanted subcutaneously into the neck area of mice. 13 mice were implanted with 5 mg pellets (5 males, 8 females), and 5 mice were implanted with 7.5 mg pellets (4 males, 1 female). The mice were anaesthetised with isofluorane. Caprofen (Caprieve Small Animal Solution for Injection, Norbrook, National Veterinary Services, Code 219129) was administered pre-operatively at 8.3 mg/kg via subcutaneous injection as analgesic. The wound at the pellet insertion site was closed with GLUture topical tissue adhesive (Zoetis, National Veterinary Services, Code 288615). Tumour development and appearance were monitored and recorded daily.

For histological analyses, the tumours were fixed overnight with 4% paraformaldehyde (Santa Cruz, cat nr sc-281692), transferred to 70% EtOH (Ethanol absolute, Sigma cat nr 32205 diluted to 70% in water) and embedded in paraffin. Samples were then cut at 8 µm. Hematoxylin and Eosin staining of paraffin-embedded tumours were performed as described previously [16].

### Results

#### K14CreER driven activation of K-ras^G12D^ and p53^LSL-R172H^ in the epidermis

**Fig. 1:**
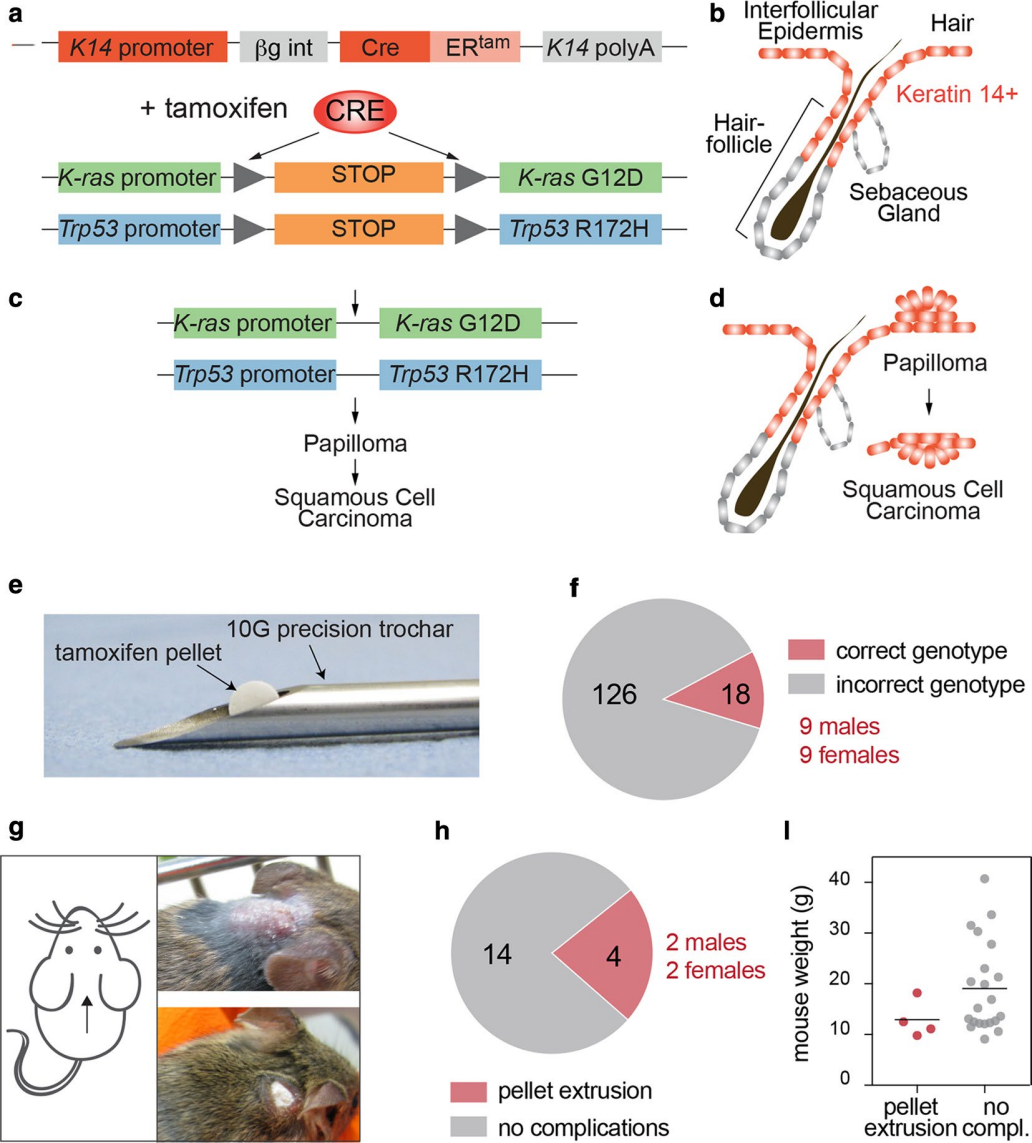
**a** Schematic representation of the keratin-14 (K14)-driven inducible Cre-recominase (Cre) and the Kras^G12D^ and p53^R172H^ transgenes prior the excision of lox-STOP-lox cassette. ßg int: Beta-globin 5’ untranslated region (UTR) and an intronic sequence. ER^tam^: tamoxifen inducible estrogen receptor. PolyA: polyadenylation signal. **b** K14-driven (red) expression in skin. **c**, **d** Transgenes after recombination (**c**) and schematic of tumour development and progression. **e** A 10 Gauge (**g**) trochar and tamoxifen pellet used for the surgeries. **f** Number of mice with a correct (red) and incorrect (grey) genotype after breeding to generate experimental cohorts. **g** Site of pellet insertion (left hand panel) and examples of successful pellet insertion (upper right hand panel) and a partially extruded pellet (lower right hand panel). **h** Number of mice showing pellet extrusion (red) in the experiment. **i** Weight of mice with extruded pellets and not extruded pellets (no complications). Lines indicate mean values

To trigger the formation of SCCs in the mouse back skin we used a Cre-recombinase inducible transgenic mouse line that carried oncogenic point mutations in the alleles of p53 (p53^LSL-R172H^) and K-ras (K-ras^LSL-G12D^) (Fig. 1a, b) [6, 15]. Skin tumour formation is initiated by activation of the endogenous K-ras^LSL-G12D^ and p53^R172H^ alleles (Fig. 1a) [13]. We activated the oncogenic alleles in the epidermis by conditionally inducing Cre-recombinase under the control of the keratin 14 (K14) promoter by administering tamoxifen (Fig. 1b) [14]. K14 (together with K5) forms the main keratin in keratinocytes in the basal, undifferentiated layer of stratified squamous epithelia that includes the skin and the inner lining of the mouth and the esophagus [17–19]. K14-driven activation of the oncogenes lead to the development of skin papillomas, a proportion of which were expected to undergo malignant conversion into invasive SCCs (Fig. 1c, d) [20].

To maintain the transgenic lines and generate experimental cohorts, the mice have to be kept as heterozygous for both K-ras^LSL-G12D^ and p53^LSL-R172H^ alleles. K-Ras^LSL-G12D^ homozygous animals lack functional K-Ras and show early embryonic lethality, and no embryos survive past E11.5 [6]. Mice homozygous for the p53^LSL-R172H^ alleles are prone to develop a variety of internal tumours within 5–6 months [21]. Thus, only K14-CreER^+^::K-ras^LSL-G12D/wt^::p53^LSL-R172H/wt^ were used for this study. Untreated control animals with this genotype showed very low levels of leakiness for the transgenes. Around 13% (2 out of 15 animals) of untreated CreER^+^::K-ras^LSL-G12D/wt^::p53^LSL-R172H/wt^ mice developed spontaneous papillomas after 16 weeks of age.

#### Intraperitoneal injections and topical application of tamoxifen to activate K-ras^G12D^ and p53^LSL-R172H^ in the epidermis

In pilot experiments, we first administered tamoxifen via intraperitoneal (IP) injection or topical application directly to a shaved area of the back skin in two small cohorts of mice (4 mice for IP and 17 mice for topical). Both methods are standard methods to apply tamoxifen and as expected, resulted in the development of skin papillomas. However, in response to both methods of tamoxifen application, all mice developed papillomas around the mouth area, which hindered them from eating. Due to weight loss, all mice in our experiments had to be culled before they developed malignant carcinomas (*data not shown*).

#### Using tamoxifen pellets to activate K-ras^G12D^ and p53^LSL-R172H^ in the epidermis

In order to minimise mouth papilloma formation, we aimed to induce the transgenes more locally and used tamoxifen pellets that can be subcutaneously inserted using a trochar (Fig. 1e). Subcutaneous pellet insertion has the advantage that it continuously releases tamoxifen over a prolonged period of time. Here, we used pellets that release a total dose of 5 or 7.5 mg of the drug over 21 days.

To start a carcinogenesis experiment we set up 15 breeding pairs and obtained a total of 144 offspring, of which 12.5% carried the right combination of transgenes (Fig. 1f). We surgically inserted the pellet into the neck area, yet in around 20% of animals, the pellet was extruded from the skin (Fig. 1g, h). Pellet extrusion was independent of the sex of the animal, but seemed to be linked to the age (size) of the animal. While it occurred in a fraction on animals with a body weight below 20 g, it never occurred in mice heavier than 20 g (Fig. 1i).

#### Mouth papilloma formation is the bottleneck of carcinogenesis experiments

**Fig. 2:**
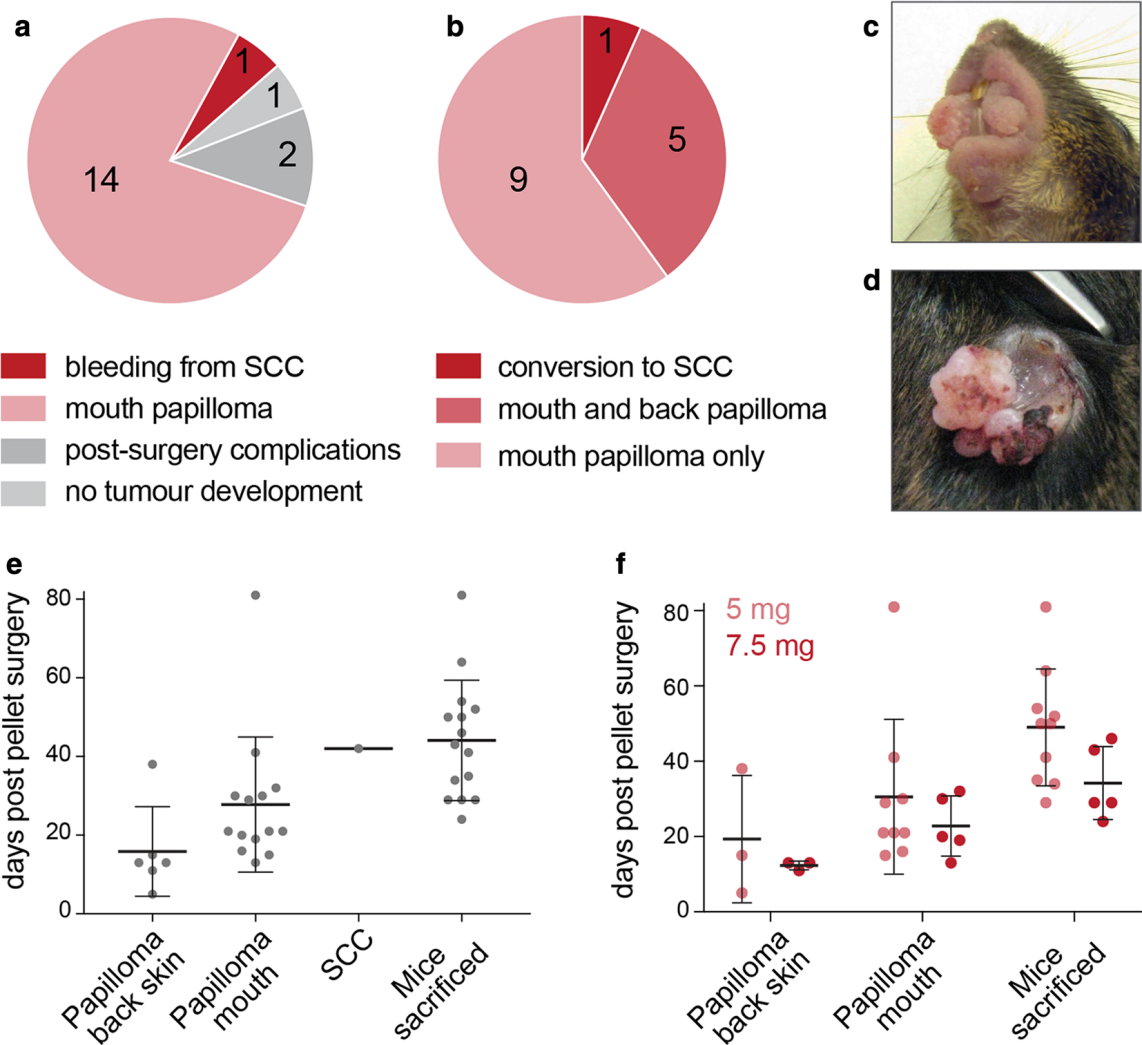
**a**, **b** Reasons for culling (**a**) and types of tumours developed after tamoxifen pellet implantation (**b**) with numbers of mice per category indicated on the graph. **c**, **d** Macroscopic appearance of a mouth papilloma (**c**) and a SCC located on back skin (**d**). **e** Timing (days post pellet insertion) of first appearance of back and mouth papillomas, conversion of one back papilloma to SCC, and the timing of tissue collection (mice sacrificed). Error bars: standard deviation of the mean. **f** The timing of first appearance of back and mouth papillomas and the timing of tissue collection (mice sacrificed) using differently dosed tamoxifen pellets. Error bars: standard deviation of the mean

15 out of the 18 experimental animals, which underwent pellet insertion surgery developed tumours (Fig. 2a). With the exception of one animal, 14 developed mouth papillomas (Fig. 2b, c). Only 6 out of the 15 animals also developed papillomas on the back skin. The back papilloma of only one mouse progressed to a SCC (Fig. 2d).

Over the course of our experiment, back skin papillomas formed first, on average on day 16 post pellet implantation (Fig. 2e). Mouth papillomas formed later, on average on day 27 post pellet implantation. Mice were maintained on average for 17 days from the first appearance of the papillomas. Then, they lost weight and needed to be culled. A standard measurement in UK home office licences is that a mouse that lost more than 15% of body weight compared to wild-type littermates must be culled. This leads to the termination of the experiment in average on day 44 post-surgery (Fig. 2e; see ‘mice sacrificed’). The single SCC conversion occurred in our model on day 42 post-surgery. Thus, the development of mouth papillomas is the bottleneck for the experimental protocol. Our experiment illustrates that due to the development of mouth papillomas, it is very challenging to maintain this particular mouse model long enough to allow for conversion to malignant SCCs.

The high incidence of mouth papilloma formation might be due small wounds caused by abrasion inside the mouth induced by solid food pellets [22]. To refine the procedure, we fed the mice with a non-solid diet. While the use of non-solid food failed to reduce mouth papilloma formation, it improved the well-being of the mice by for instance reducing bleeding from the papillomas.

Finally, we tested whether higher doses of tamoxifen enhanced formation of SCC. We used two types of tamoxifen pellets: a total dose of 5 mg or 7.5 mg released over 21 days. The increase of tamoxifen dose from 5 to 7.5 mg slightly sped up the formation of all types of tumours, yet the difference was not statistically significant. The higher dose of tamoxifen did not affect the formation of mouth papillomas (Fig. 2f).

#### Conversion rate of papillomas to SCCs is low

**Fig. 3:**
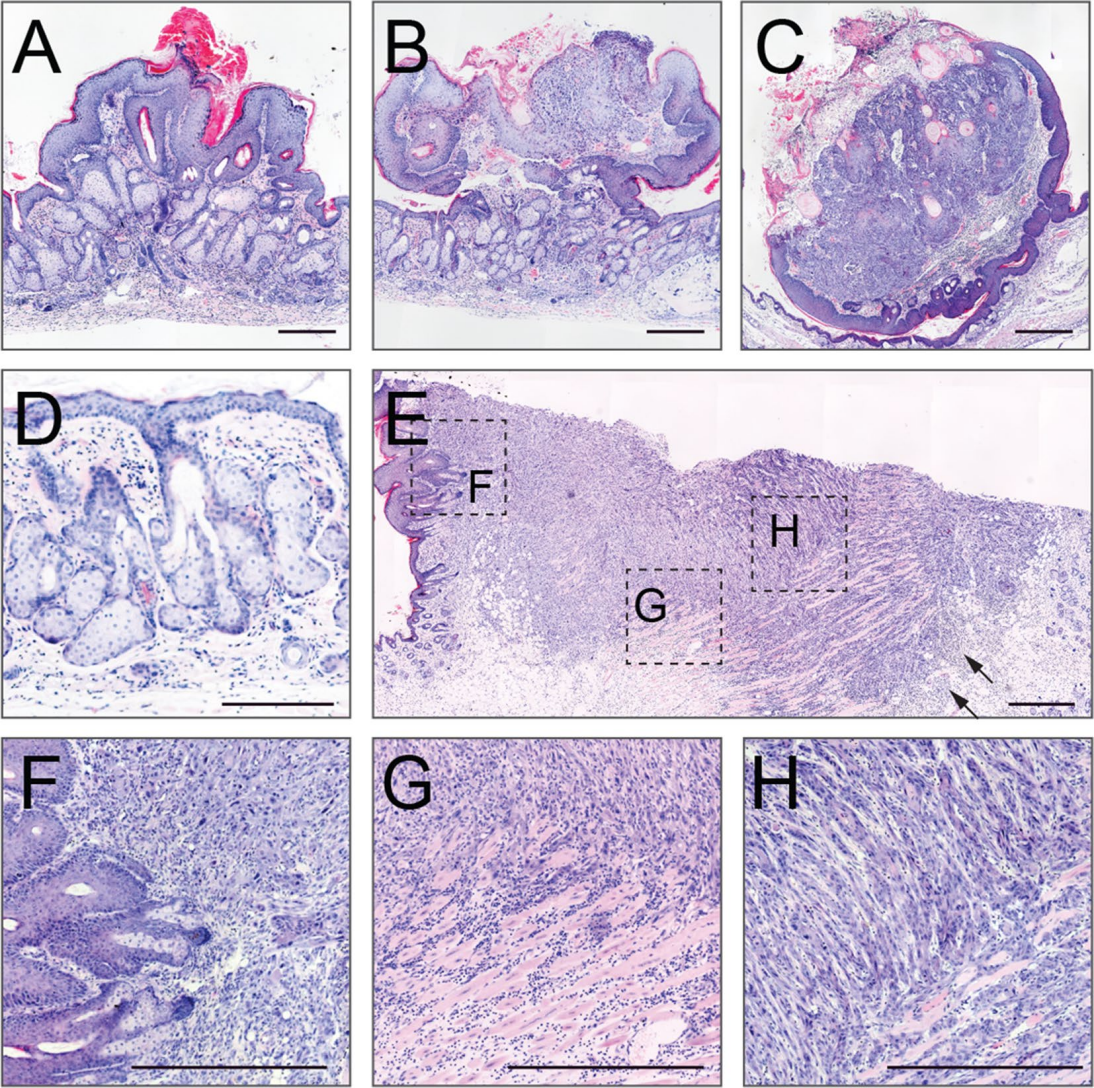
**A**–**C** Representative examples of Hematoxylin and Eosin stained sections of skin papillomas. **D** Hematoxylin and Eosin stained sections of back skin showing enlarged sebaceous glands. **E**–**H** Histology of sectioned malignant SCC from back skin. F, G and H show magnified areas as highlighted in **E** by the black boxes. Scale bar (**A**–**C**, **E**–**H**): 500 µm; Scale bar (**D**): 200 µm

Histological section of typical papillomas and the SCC are shown in Fig. 3A–C, E–H. A common phenotype in response to the oncogenic activation in mouse epidermis is hyperplasia of cells in the stratum basale and the sebaceous glands (Fig. 3D), which can be explained by the activation of a single mutant *Kras*^*G12D*^ allele [23]. The carcinoma showed neoplastic regions with an invasive front towards the deeper tissues, which are common characteristics of a SCC (Fig. 3E–H). In addition, we detected inflammation and dermal fibroblast proliferation (desmoplasia) (Fig. 3E; arrows). Neoplastic keratinocytes are polygonal, lack polarity and show anisocytosis and anisokaryosis (Fig. 3H). Together, one out of 15 (7%) papillomas progressed to a malignant SCC.

## Limitations

In this study we highlight some limitations when using the K14-CreER::K-ras^LSL-G12D^::p53^LSL-R172H^ genetic mouse model to study the progression from benign papillomas to malignant carcinomas in skin.

Generating the mice by crossing requires in average 1 year, given that experimental mice carry at least the following alleles: p53^LSL-R172H^, K-ras^LSL-G12D^, an inducible Cre-recombinase, and often an additional transgene of interest. Breeding the mice to generate the experimental cohorts required a large number of mice. Here, we bred 144 animals to generate 18 experimental mice carrying the required transgenes. Due to the genetic crossing strategy and low tumour progression frequencies, less than 1% of mice originally set up for the experiment were used for further studies.

Combining the point mutant alleles p53^LSL-R172H^ and K-ras^LSL-G12D^ has been shown to increase skin tumour formation, accelerate tumour progression, and induce metastasis when compared with single deletion of p53 or over-expression of K-ras [13]. Given that both p53^LSL-R172H^ and K-ras^LSL-G12D^ alleles need to be heterozygous, the yield of experimental animals with correct genotype is low. A possible solution might be using different conditional alleles of oncogenic H-Ras or N-Ras and p53 that can be maintained as homozygous [24, 25]. Using these alleles would increase the number of study animals and the number of malignant SCCs. However, whether the use of different alleles enhances the progression rate to malignant SCCs after activation with tamoxifen remains to be determined.

The main limitation for our study was however, that K14 promoter-driven expression of oncogenic *K*-*Ras* and *p53* leads to the formation of mouth papillomas in the vast majority of experimental animals. These benign tumours interfere with food intake and animal health, resulting in culling the animals before the onset of SCC formation on the back skin. The use of alternative promoters to drive oncogenic driver mutations in different cell lineages might be a solution to induce the tumour formation at a different location.

## Abbreviations

SCC: squamous cell carcinomas
K14: keratin 14
Cre: Cre recombinase
ER: estrogen receptor
K5: keratin 5
wt: wild-type
IP: intraperitoneal
